# 1561. Efficacy of Bictegravir/Emtricitabine/Tenofovir Alafenamide (B/F/TAF) Versus Dolutegravir (DTG)-Based 3-Drug Regimens in Adults With HIV Who Have Suboptimal Antiretroviral Adherence

**DOI:** 10.1093/ofid/ofad500.1396

**Published:** 2023-11-27

**Authors:** Kristen Andreatta, Paul E Sax, David Alain Wohl, Michelle L D’Antoni, Hailin Huang, Jason Hindman, Christian Callebaut, Hal Martin

**Affiliations:** Gilead Sciences, Inc., Foster City, California; Brigham and Women’s Hospital, Harvard Medical School, Boston, Massachusetts; UNC School of Medicine, Chapel Hill, NC; Gilead Sciences, Inc., Foster City, California; Gilead Sciences, Inc., Foster City, California; Gilead Sciences, Foster City, California; Gilead Sciences, Foster City, California; Gilead Sciences, Foster City, California

## Abstract

**Background:**

Adherence to daily oral antiretroviral therapy is important for sustaining HIV suppression. B/F/TAF Studies 1489, 1490, 4458, 1844 and 4030 demonstrated the noninferior efficacy of B/F/TAF versus DTG + 2 nucleoside reverse transcriptase inhibitors (NRTIs). We retrospectively assessed drug adherence and effect on virologic outcomes.

**Methods:**

All studies were double-blind, placebo-controlled, and enrolled treatment-naïve (1489, 1490, 4458) or virologically suppressed (1844, 4030) adults. Participants were randomized 1:1 to receive B/F/TAF or DTG + 2 NRTIs **(Table)** plus placebo; as a result, all received multiple tablets. Participants with ≥ 1 returned pill bottle and ≥ 1 on-treatment HIV-1 RNA measurement were included in the analysis. Adherence was calculated by pill count; virologic outcome was assessed by last on-treatment HIV-1 RNA.

**Figure:**
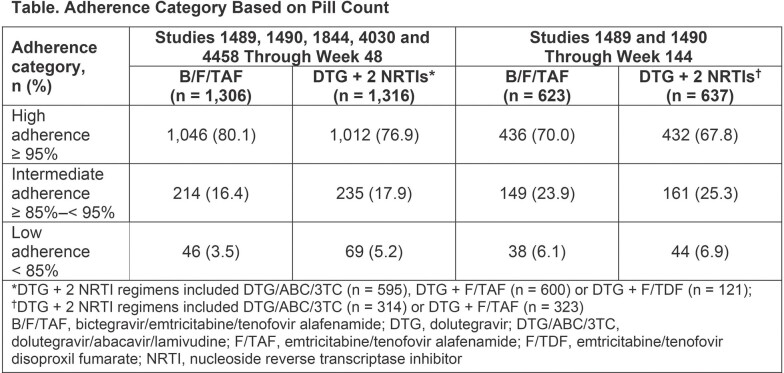

**Results:**

Altogether, 2,622 participants were included (B/F/TAF: 1,306; DTG + 2 NRTIs: 1,316). The proportions of participants with high (≥ 95%), intermediate (≥ 85%‒< 95%) or low (< 85%) adherence were similar between the 2 groups; few had low adherence (B/F/TAF: 46 [3.5%]; DTG + 2 NRTIs: 69 [5.2%] through Week [W] 48). Overall, 98.5% (n=1,287) in the B/F/TAF group and 98.2% (n=1,292) in the DTG + 2 NRTI group had virologic suppression (HIV-1 RNA < 50 copies/mL) at last on-treatment visit through W48. In the B/F/TAF group, virologic suppression was similar in those with high and intermediate adherence versus those with low adherence; however, in the DTG + 2 NRTI group, virologic suppression was significantly higher in those with high and intermediate adherence compared with low adherence (*P* ≤ 0.002, **Figure**). Similar results were observed at W144 in 2 studies (1489, 1490) with additional follow-up data. Nine participants with low adherence had HIV-1 RNA ≥ 50 copies/mL at their last visit through W48: 3 subsequently resuppressed (B/F/TAF: 1; DTG + 2 NRTIs: 2), 5 discontinued (all DTG + 2 NRTIs) and 1 was lost to follow-up (B/F/TAF).

**Figure:**
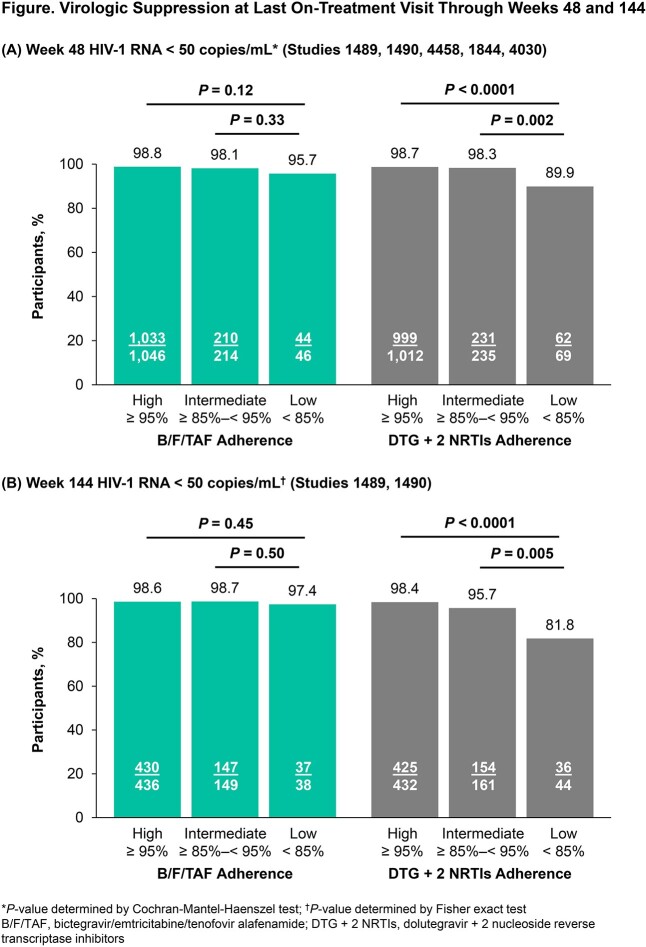

**Conclusion:**

Overall, most participants receiving either placebo-controlled B/F/TAF or DTG + 2 NRTIs demonstrated ≥ 85% adherence. In those with suboptimal adherence, B/F/TAF treatment maintained high levels of virologic suppression, while those with suboptimal DTG + 2 NRTI adherence had reduced virologic suppression.

**Disclosures:**

**Kristen Andreatta, MS**, Gilead Sciences, Inc.: Employee|Gilead Sciences, Inc.: Stocks/Bonds **Paul E. Sax, MD**, Gilead: Advisor/Consultant|Gilead: Grant/Research Support|Janssen: Advisor/Consultant|Merck: Advisor/Consultant|ViiV: Advisor/Consultant|ViiV: Grant/Research Support **David Alain Wohl, MD**, Gilead: Advisor/Consultant|Gilead: Grant/Research Support|Gilead: Honoraria|Janssen: Advisor/Consultant|Janssen: Honoraria|Theratech: Advisor/Consultant|Theratech: Honoraria|ViiV: Advisor/Consultant|ViiV: Grant/Research Support|ViiV: Honoraria **Michelle L. D’Antoni, PhD**, Gilead: Employment|Gilead: Stocks/Bonds **Hailin Huang, PhD**, Gilead: Employment **Jason Hindman, PharmD, MBA**, Gilead: Employment|Gilead: Stocks/Bonds **Christian Callebaut, PhD**, Gilead: Employment **Hal Martin, MD**, Gilead: Employment

